# Computed Tomography Bronchus Sign Subclassification during Radial Endobronchial Ultrasound-Guided Transbronchial Biopsy: A Retrospective Analysis

**DOI:** 10.3390/diagnostics13061064

**Published:** 2023-03-10

**Authors:** Tatsuya Imabayashi, Yuji Matsumoto, Keigo Uchimura, Hideaki Furuse, Takaaki Tsuchida

**Affiliations:** 1 Department of Endoscopy, Respiratory Endoscopy Division, National Cancer Center Hospital, 5-1-1 Tsukiji, Chuo-ku, Tokyo 104-0045, Japan; 2 Department of Thoracic Oncology, National Cancer Center Hospital, Tokyo 104-0045, Japan

**Keywords:** bronchoscopy, bronchus sign, peripheral pulmonary lesion, radial endobronchial ultrasound, lung cancer

## Abstract

The presence of computed tomography bronchus sign (CT-BS) substantially increases the diagnostic yield of peripheral pulmonary lesions. However, the clinical significance of subdividing CT-BS remains controversial. We classified bronchus types on CT into six subtypes (CT-BS group I: types Ia–Ic with the bronchus connected within the lesion, group II: types IIa–IIc without connection) to clarify the differences in their characteristics and investigate the factors associated with diagnosis during radial endobronchial ultrasound (rEBUS)-guided bronchoscopy. In total, 1021 cases were analyzed. Our findings in diagnostic yields were that in CT-BS group I, penetrating type Ic was inferior to obstructed type Ia and narrowing type Ib (59.0% vs. 80.0% and 76.3%, *p* < 0.001, *p* = 0.004); in CT-BS group II, compressed type IIa showed no difference when compared with invisible type IIb and uninvolved type IIc (IIa: 52.8% vs. IIb: 46.3% and IIc: 35.7%, *p* = 0.253). Multivariable analysis revealed that bronchus type (types Ia and Ib vs. Ic) was a significant independent predictor of successful diagnosis in CT-BS group I (odds ratio, 1.78; 95% confidence interval, 1.04–3.05; *p* = 0.035), along with known factors such as rEBUS visualization. CT-BS subclassification may provide useful information regarding the bronchoscopic technique to facilitate accurate diagnosis.

## 1. Introduction

With advances in diagnostic imaging and increased use of low-dose helical computed tomography (CT) in lung cancer screening [1], peripheral pulmonary lesions (PPLs) are a common respiratory disease detected routinely. However, their diagnosis remains a challenge. Over the past two decades, various bronchoscopic techniques for the diagnosis of PPLs emerged, including radial endobronchial ultrasound (rEBUS), virtual bronchoscopic navigation, guide sheath (GS), ultrathin bronchoscopy, and electromagnetic navigation bronchoscopy (ENB) [2,3,4,5,6,7,8]. Nevertheless, the diagnostic yield of PPLs remains suboptimal compared with that of transthoracic needle aspiration (TTNA) and is collectively around 70%, varying considerably among different studies [2].

Several reports demonstrated that diagnostic yield, regardless of technique, is affected by CT characteristics of the target lesion, such as size, distance from the hilum, and presence of CT bronchus sign (CT-BS) [2,8,9,10,11,12,13,14,15,16,17]. The CT-BS, which indicates the presence of a bronchus leading directly to PPLs, is derived from the Tsuboi classification [18]. The Tsuboi classification originally comprised four types of anatomical relationships between the bronchi and surgically resected tumors and was used to determine the optimal transbronchial biopsy (TBB) approach. Types I (the patent bronchus leads directly to the tumor) and II (the bronchus is contained in the tumor) are favorable for TBB. However, types III (the bronchus is compressed and narrowed by the tumor) and IV (the proximal bronchus is very narrowed by peribronchial-submucosal spread of the tumor or by enlarged lymph nodes) are difficult to diagnose because the bronchial mucosa is intact or the forceps cannot reach the tumor, respectively [19].

As the Tsuboi classification is not fully applicable to the relationship between the bronchi and PPLs on CT, more practical CT-BS subclassifications were proposed [10,20,21]. However, eventually, a simple classification into two categories, positive or negative CT-BS, became widely favored. The presence of CT-BS substantially increases the diagnostic yield through the probability of reaching the lesion, that is, rEBUS visualization yield [14]. A meta-analysis of nine studies investigating the utility of rEBUS reported that the weighted diagnostic yields for PPLs with and without CT-BS were 76.5% (95% confidence interval [CI], 65.9–85.6%) and 52.4% (95% CI, 37.6–67.0%), respectively [22]. Nevertheless, the definition of CT-BS is ambiguous and often confusing. Although the four types from the Tsuboi classification were previously considered as positive CT-BS [23,24], this definition changed in recent studies, with the development of thinner CT sections [10,15,16,21].

To date, most studies focused on comparing the presence and absence of CT-BS. Moreover, reports regarding rEBUS visualization and diagnostic yields for each bronchus type are scarce and were limited to relatively small cohorts. Bronchus types can be classified into the following six types: the four types from the Tsuboi classification, a bronchus type in which the bronchus is not involved in the PPL (originally considered negative CT-BS), and a bronchus type in which the dilated bronchus penetrates the PPL with intact mucosa [10,18,19,20,21,25]. Investigating rEBUS visualization and diagnostic yields of these six types in a larger cohort would be helpful in further interpreting CT-BS. This penetrating type has not yet been investigated in terms of differences in diagnostic outcomes from Tsuboi types I and II and appropriate bronchoscopic sampling methods.

Therefore, this study aimed to identify the lesion characteristics of each bronchus type and to investigate the factors associated with successful diagnosis of PPLs in rEBUS-guided TBB (rEBUS-TBB).

## 2. Materials and Methods

### 2.1. Patients

This was a retrospective study of consecutive patients who underwent rEBUS-TBB for PPLs between November 2015 and October 2017 at the National Cancer Center Hospital, Tokyo, Japan. Patients with central pulmonary lesions with evidence of endobronchial involvement, those who underwent re-examination for the same lesion, those with unknown final diagnosis due to loss at follow-up, and those with lack of evaluation by high resolution CT (HRCT) imaging of lesions that were <1 mm in thickness were excluded. The study was conducted in accordance with the Declaration of Helsinki. This study was approved by the National Cancer Center Institutional Review Board (No. 2018-090). The requirement for informed consent was waived due to the retrospective nature of the study.

### 2.2. Bronchus Type Classification

Two pulmonologists (T.I. and K.U.), blinded to the final diagnosis, retrospectively reviewed HRCT images of target lesions that were <1.0 mm in thickness and obtained at least 1 month before bronchoscopy. Final decisions on the classification were reached by consensus. Initially, if the bronchus was leading to the target lesion, the lesion was classified as CT-BS group I; other cases were classified as group II. Subsequently, the relationship between the lesion and nearest bronchus and/or artery was classified into six bronchus subtypes based on previous reports [10,18,19,20,21,25].

**Figure 1:**
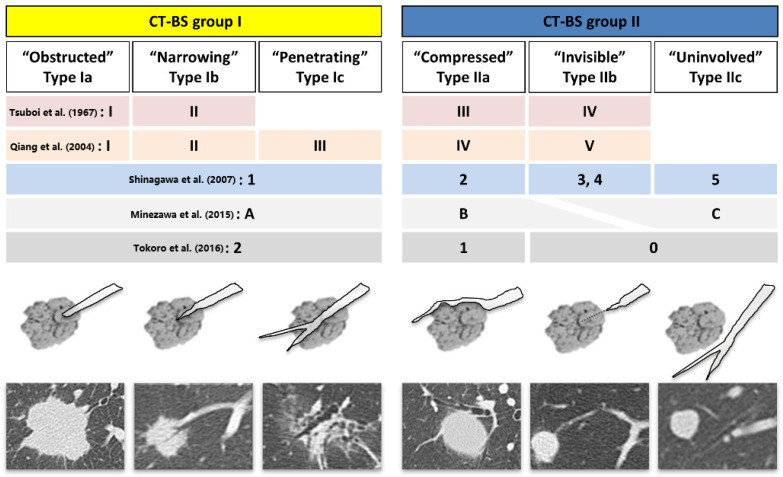
Schematic of classification of bronchus types, integrated with previously reported classifications. CT-BS, computed tomography bronchus sign [10,18,20,21,25].

As shown in Figure 1, the responsible bronchus that directly reached within the target lesion (CT-BS group I) was obstructed, narrowing, and penetrating in types Ia, Ib, and Ic, respectively. When multiple bronchus types reached within the lesion but no dominant bronchi could be unequivocally identified, types Ia, Ib, and Ic were preferentially selected, in that order. Of those classified as CT-BS group II, the bronchus was compressed with a narrow lumen near the edge of the target lesion in type IIa, the pulmonary artery was leading to the lesion but the responsible bronchus was not clearly visible or not traceable continuously in type IIb, and neither the bronchus nor pulmonary artery was involved in the lesion in type IIc.

### 2.3. Procedures

All procedures were performed under local anesthesia and conscious sedation. A bronchoscope with a 2.0 mm working channel (BF-P260F, BF-P290, or BF-260; Olympus, Tokyo, Japan) or a 2.8 mm working channel (BF-1T260; Olympus) was advanced toward the target lesion through the bronchus, with reference to the virtual bronchoscopic navigation (Ziostation2, Ziosoft Ltd., Tokyo, Japan). A 1.4- or 1.7 mm rEBUS probe (UM-S20-17S or UM-S20-20S; Olympus) was subsequently advanced toward the lesion along with or without a 1.95- or 2.55 mm GS (SG-200C or SG-201C; Olympus) under fluoroscopic guidance (VersiFlex VISTA; Hitachi, Japan). The obtained rEBUS findings were classified into “within”, “adjacent to”, or “invisible” according to previous reports [3]. Once the target lesion was visualized by rEBUS, the rEBUS probe was withdrawn and sampling was conducted at the same site. TBB was performed with a 1.5- or 1.9 mm forceps (FB-233D or FB-231D; Olympus), depending on the technique (the former for thin GS and the latter for thick GS or non-GS). For cases with rEBUS findings classified as “adjacent to” or “invisible”, transbronchial needle aspiration (TBNA) was performed using a 21-gauge aspiration needle (NA-1C-1; Olympus) before TBB, if technically feasible and safe [26]. Decisions on these procedures, including selection of appropriate sampling tools (i.e., forceps, brush, needle, or lavage) and biopsy number, were made at the discretion of the examiner. Bronchoscopy was performed mostly in the outpatient setting, and patients were observed in the recovery room for 2 h after the procedure until they were discharged. Chest radiographs were acquired only if the patient complained of symptoms suggestive of pneumothorax.

### 2.4. Diagnosis

The final diagnoses were established on the basis of pathologic evidence, microbiological analyses, or clinical follow-up. PPLs were considered malignant if the initial biopsy was negative for malignancy and repeated biopsies using any biopsy method revealed malignancy. PPLs were considered as “suspected malignancy” if the biopsies were negative but radiological follow-up confirmed disease progression without histological confirmation, and cancer treatment (e.g., radiation therapy for undiagnosed PPLs) was initiated. Benign diagnoses, which could not be determined pathologically or microbiologically, were confirmed by radiological and clinical follow-up at least 1 year after the procedure.

Bronchoscopy was considered diagnostic if the collected samples exhibited malignancy microbiologically or specific benign findings (such as granuloma or organizing pneumonia) with reasonable radiologic and clinical findings. Biopsies exhibiting “fibrosis or inflammation” were also considered diagnostic if they demonstrated radiological and clinical improvement or were consistent with the final diagnosis from subsequent surgical biopsies. Bronchoscopy was considered non-diagnostic in the absence of these evidences. Non-diagnostic biopsies included inadequate samples (e.g., normal lung or peribronchial tissue) or biopsies with a different diagnosis on follow-up examination.

### 2.5. Data Collection and Statistical Analyses

Electronic medical records were analyzed to collect data on relevant aspects, including patient and lesion characteristics and procedural details of bronchoscopy, as follows: sex, age, lesion size, lesion location (lobe or distance from the hilum or visceral pleura), lesion appearance on CT, visibility on fluoroscopy (frontal and oblique images), bronchoscopic and final diagnoses, rEBUS findings, use of GS, forceps size, TBNA, brushing, bronchial lavage, and procedure-related major complications. The lesion location from the hilum was classified into two groups: “inner” for lesions in the inner and middle third ellipses, and “outer” for lesions in the outer third ellipse [27]. The locational relationship with pleura was classified into two categories: apart from or abutting on the pleura. rEBUS visualization and diagnostic yields of rEBUS-TBB in CT-BS groups I and II were evaluated. rEBUS visualization yield was defined as “cases successfully detected within or adjacent to the lesion by rEBUS divided by total cases”. Diagnostic yield was defined as “diagnostic cases divided by total cases”.

A major complication was defined as an event that necessitated premature termination of the procedure or symptomatic postprocedural sequela, including pneumothorax, hemorrhage, infection, or another untoward life-threatening outcome [28,29].

Data analyses were performed using EZR software (Saitama Medical Center, Jichi Medical University, Saitama, Japan). Categorical variables were analyzed using the Pearson’s chi-square test or Fisher’s exact test. Continuous variables were analyzed using the Mann–Whitney *U* test or Kruskal–Wallis test. The patient and lesion characteristics and procedural details for each bronchus type were analyzed. For each bronchus type, the diagnostic yields per “within” and “adjacent to” rEBUS finding and per 1.5- and 1.9 mm forceps were evaluated. Univariable and multivariable (variables with *p* < 0.1 were entered) logistic regression analyses were performed to identify the factors affecting diagnosis in CT-BS groups I and II, with regard to previously reported clinically important factors [7,8,9,10,13,14,15,20]. Odds ratios (ORs) and associated 95% CIs were calculated to assess the contribution of significant factors. *p*-values were two-sided, and *p* < 0.05 was considered statistically significant. For three paired comparisons, the *p*-values were corrected using the Bonferroni method.

## 3. Results

**Figure 2:**
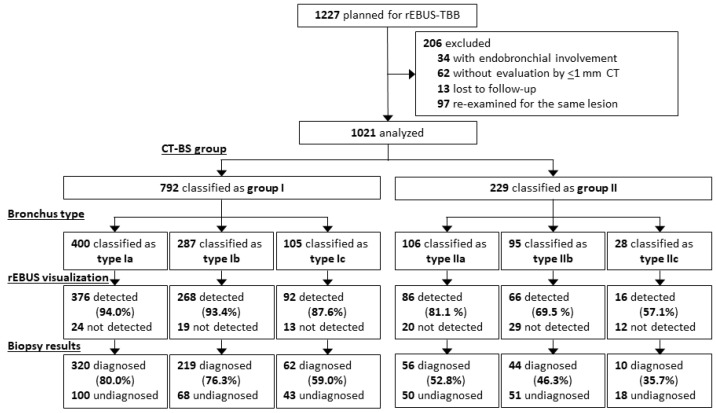
Study cohort flowchart. rEBUS-TBB, radial endobronchial ultrasound guided transbronchial biopsy; CT, computed tomography; CT-BS, CT bronchus sign.

A total of 1021 patients, including 792 (77.6%) in CT-BS group I and 229 (22.4%) in CT-BS group II, were analyzed (Figure 2). CT-BS group I was classified into types Ia, Ib, and Ic (comprising 400, 287, and 105 patients, respectively); CT-BS group II was classified into types IIa, IIb, and IIc (comprising 106, 95, and 28 patients, respectively). Patient and lesion characteristics of CT-BS groups I and II are presented in Table 1 and Table 2, respectively. Compared with CT-BS group II, group I contained more lesions that were larger (median diameter of 25.7 mm vs. 15.6 mm, *p* < 0.001) and visible on fluoroscopy (76.9% vs. 54.3%, *p* < 0.001). Intra-group comparisons revealed that type Ic was smaller in size (median of 20.0 mm), less visible on fluoroscopy (59.0%), more distant from the pleura (85.7%), and contained more ground-glass nodules (74.3%) compared with types Ia and Ib. Type IIa had the largest size (median of 18.6 mm) and greatest number of visible lesions on fluoroscopy (64.2%), type IIb had the greatest number of outer lesions (85.3%) and lesions abutting the pleura (48.4%), and type IIc had the lowest frequency (12.2%). Malignant lesions comprised 83.3% and 78.6% of lesions in CT-BS groups I and II, respectively. The final diagnoses for each group are presented in Table S1 (see Supplementary Material).

Procedural details for each bronchus type are presented in Table 3. The rEBUS visualization yield (92.9% vs. 73.4%, *p* < 0.001) and yield of “within” finding (59.0% vs. 28.8%, *p* < 0.001) were higher in CT-BS group I than in group II. Within each group, type Ic had the lowest rEBUS visualization yield of 87.6% but not significant (vs. type Ia: *p* = 0.102, vs. type Ib: *p* = 0.279). In contrast, type IIa had the highest visualization yield of 81.1% (vs. type IIb: *p* = 0.210, vs. type IIc: *p* = 0.036) and contained many “adjacent to” rEBUS findings (57.5%). The proportions of non-GS method and use of a 1.9 mm forceps increased in the order of type Ia, Ib, and Ic (22.8% and 44.8% in type Ia, 32.1% and 75.3% in type Ib, 46.7% and 86.7% in type Ic). In CT-BS group II, the non-GS method was selected in half or more. In more peripherally located type IIb, a 1.5 mm forceps was used in 45.3% along with a thin GS, and TBNA was performed in only 21.1%. On the other hand, TBNA was performed in approximately half of types IIa and IIc (50.0% and 46.4%, respectively).

Factors affecting successful diagnosis in CT-BS group I were investigated (Table 4). Comparison of diagnostic yield for each bronchus type revealed no difference between types Ia and Ib (*p* = 0.260); however, the yield was higher than that for type Ic (vs. type Ia: *p* < 0.001, vs. type Ib: *p* = 0.003). Univariable analysis revealed that in addition to bronchus type (types Ia and Ib vs. Ic), lesion size, location from the hilum, lesion appearance on CT, visibility on fluoroscopy, rEBUS detection, and use of GS were significantly associated with the diagnosis. In multivariable analysis, rEBUS detection was the most significant factor affecting successful diagnosis (OR, 4.86; 95% CI, 2.58–9.14; *p* < 0.001). Bronchus type (types Ia and Ib vs. Ic) was a significant independent predictor of diagnosis (OR, 1.78; 95% CI, 1.04–3.08; *p* = 0.035), along with rEBUS detection, visibility on fluoroscopy, lesion size, and use of GS.

In CT-BS group II, the overall diagnostic yield was 48.0%, with no significant difference between each bronchus type (*p* = 0.253) (Table 5). In univariable analysis, lesion appearance on CT, visibility on fluoroscopy, bronchus type (type IIa vs. IIb and IIc), and rEBUS visualization affected successful diagnosis. Multivariable analysis revealed that these factors, other than bronchus type, were independently associated with the diagnosis.

**Figure 3:**
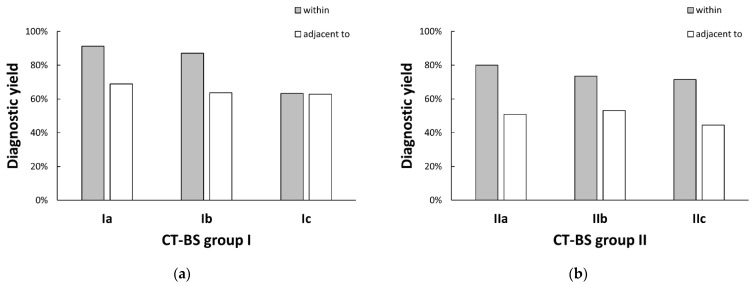
Diagnostic yields of each rEBUS finding in CT-BS groups (**a**) I and (**b**) II. In cases with a “within” image, type Ic had a lower diagnostic yield compared with types Ia and Ib (*p* < 0.001 and <0.001, respectively). In cases with an “adjacent to” image, there was no difference in diagnostic yield between the bronchus types in CT-BS group I (*p* = 0.621). In CT-BS group II, there was no difference in diagnostic yields of “within” and “adjacent to” images between the bronchus types (*p* = 0.774 and 0.955, respectively). rEBUS, radial endobronchial ultrasound; CT-BS, computed tomography bronchus sign.

The diagnostic yield of each rEBUS finding in groups I and II are shown in Figure 3a,b, respectively. In cases with a “within” image, type Ic had a lower diagnostic yield compared with types Ia and Ib (*p* < 0.001 and <0.001, respectively). In cases with an “adjacent to” image, there was no difference in diagnostic yield between the bronchus types in CT-BS group I (*p* = 0.621). In CT-BS group II, there was no difference in diagnostic yields of “within” and “adjacent to” images between the bronchus types (*p* = 0.774 and 0.955, respectively).

**Figure 4:**
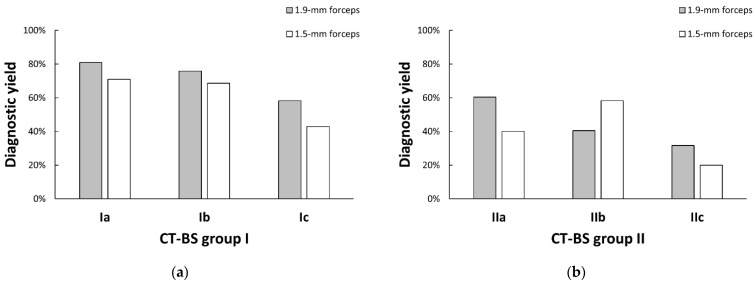
Diagnostic yields of each forceps in CT-BS groups (**a**) I and (**b**) II. The difference in diagnostic yield between the 1.9- and 1.5 mm forceps was the greatest in group Ic but not significant in CT-BS group I (58.2% [53 of 91] vs. 42.9% [6 of 14], *p* = 0.387). Compared with other bronchus types, 1.5 mm forceps cases had a higher diagnostic yield than 1.9 mm forceps cases in type IIb (58.1% [25 of 43] vs. 40.4% [19 of 47], *p* = 0.139). CT-BS, computed tomography bronchus sign.

The diagnostic yield of each forceps in CT-BS groups I and II are shown in Figure 4a,b, respectively. The difference in diagnostic yield between the 1.9- and 1.5 mm forceps was the greatest in group Ic but not significant due to the small sample size of 1.5 mm forceps cases in CT-BS group I (58.2% [53 of 91] vs. 42.9% [6 of 14], *p* = 0.387). Compared with other bronchus types, 1.5 mm forceps cases had a higher diagnostic yield than 1.9 mm forceps cases in type IIb (58.1% [25 of 43] vs. 40.4% [19 of 47], *p* = 0.139).

Overall, 9 (0.9%) patients presented with pneumothorax, among whom five (0.5%) required chest tube drainage and twenty-eight (2.7%) developed pulmonary infection. No other major complications, including severe bleeding, were observed.

## 4. Discussion

In this study, we classified bronchus subtypes on CT into six subtypes in 1021 patients with PPLs who underwent rEBUS-TBB to clarify the differences in their characteristics and investigated the factors associated with successful diagnosis. Multivariable logistic regression analysis revealed that penetrating type Ic was an independent factor influencing the diagnostic outcome in CT-BS group I, along with previously known factors such as rEBUS visualization, lesion size, visibility on fluoroscopy (pre-procedural chest radiography is often used instead), and use of GS [7,8,9,10,13,14,15,20].

In previous studies, CT-BS group I cases were classified into one to three types, as shown in Figure 1. Tsuboi types I and II corresponded to bronchus types Ia and Ib, respectively, in this study [18,19]. Unlike type Ia, type Ib is characterized by the presence of intratumoral air bronchograms, comprising a high frequency of lepidic tumor growth [30]. An air-containing bronchus that predominantly penetrates into PPLs without narrowing of the lumen was classified as our proposed type Ic. Type Ic was also characterized by ground-glass nodules accounting for >70%, <20 mm in size, and invisible on fluoroscopy in over half of them, making rEBUS visualization and diagnosis challenging.

The overall diagnosis yield for CT-BS group I in our study was 75.9%, comparable to the results of a meta-analysis [22]. Similar to this study, a previous study reported that bronchus types Ia and Ib had good diagnostic outcomes using ENB, with no difference between types [16]. However, in type Ic, as represented by ground-glass nodules, the bronchus penetrated into the tumor without mucosal invasion [25], making it challenging to obtain sufficient samples for diagnosis relative to the other types, despite confirmation of “within” images by rEBUS. Accordingly, for the diagnosis of type Ic lesions, the use of sampling devices that can obtain larger tissue samples beyond the peripheral bronchial wall is recommended [8,31]. However, the 1.9 mm forceps predominantly used in this study did not provide a sufficient diagnostic outcome. In this regard, cryoprobes permitting deep biopsies of the entire circumference may be a preferable solution, although bleeding is a greater concern for this approach than for forceps biopsy [13]. Cryobiopsy is reportedly useful in cases for which it is difficult to obtain tissue by forceps biopsy due to air space dilation within the tumor [32]. An intratumoral dilated bronchus of this type is referred to as open-bronchus sign, which is directly connected to the central airway and does not act as a tamponade against bleeding. This type was identified as a significant risk factor for TTNA-related hemoptysis [33]. Continued discussion is needed to resolve the dilemma often faced by pulmonologists in deciding between TTNA or bronchoscopy while balancing the risk of complications and diagnostic performance.

In CT-BS group II, a solid lesion was an independent factor affecting diagnostic success, in addition to rEBUS and fluoroscopic visualization as in group I. This was similar to that reported in a previous study [34]. CT-BS group II was classified into two or four types in the literature. Despite minor differences in classification, Tsuboi type III, CT-BS 1 reported by Tokoro et al., type B reported by Minezawa et al., and CT signs type 2 reported by Shinagawa et al. corresponded to bronchus type IIa in this study [10,18,19,20,21]. This type is typically classified as positive CT-BS [10,16,21,23,32], while studies focusing on negative CT-BS are considered negative [34,35], with a diagnostic yield ranging from 37.9% to 74.1%. Among CT-BS group II types, type IIa has a higher rEBUS visualization yield [20,34]; however, it contains many “adjacent to” images with comparable diagnostic performance relative to other types [36] or intermediate to CT-BS group I [20,21].

Type IIb consists of the most peripherally located lesions in CT-BS group II. As the responsible bronchus is not traceable on CT, the pulmonary artery leading to the lesion is traced instead, corresponding to CT signs types 3 and 4, termed “CT-artery sign” and Tsuboi type IV [10]. This “follow the vessel approach” is considered useful in ENB, robotic assisted bronchoscopy (RAB), and CT-guided ultrathin bronchoscopy which can reach such peripheral areas [37,38]. This study did not demonstrate a statistically significant diagnostic advantage of a 1.5 mm forceps over a 1.9 mm forceps in type IIb. Whether the reachability and sampling accuracy of 1.5 mm forceps and GS in peripheral areas are more important for successful diagnosis than the amount of tissue collected with 1.9 mm forceps needs to be validated in larger studies. On the other hand, the fluoroscopy-guided rEBUS-TBB showed no significant difference in rEBUS visualization and diagnostic yields between types IIb and IIc, in which neither the bronchus nor artery led to the lesion.

Previous reports classified positive and negative CT-BS into two categories: types Ia-Ic/IIa and IIb/IIc, respectively [10,16,21,23,32] or group I and group II [34,35]. Based on the results of this study, it would be appropriate to classify CT-BS positive and negative into two groups I and II. As there was no statistical difference in the diagnostic yield of types Ic and IIa (*p* = 0.406), CT-BS could also be further subdivided into groups A (types Ia/Ib), B (types Ic/IIa), and C (types IIb/IIc).

We highlighted some values of CT-BS subclassification in peripheral diagnosis during rEBUS-TBB. Subdividing CT-BS allows for more accurate prediction of rEBUS findings [20]. In addition, this study revealed that the diagnostic yield of “within” images by rEBUS, a strong predictor for successful diagnosis, differs according to bronchus type. These findings will guide pulmonologists in determining the appropriate sampling devices such as selecting the GS size, adding TBNA, or switching to other methods, including the non-GS method. This should consider not only diagnostic performance, but also the cost and amount of tissue samples required for molecular analysis in advanced lung cancer [39].

This study had some limitations. First, this was a retrospective study conducted at a single center. Diagnostic outcomes may vary by technique, institution, and proportion of the malignant disease. Moreover, bronchus type classification may vary by reviewer and CT imaging conditions. In this study, two expert pulmonologists sufficiently familiar with this classification method reviewed the images; however, the interobserver agreement was not investigated. The interobserver agreement regarding CT-BS reported by Minezawa et al. [20] was moderate (Fleiss’s κ: 0.559) in 109 PPLs without CT-BS, as described by Hong et al. [34]. Second, the study may have been subject to selection bias. Although this study was conducted using a relatively large cohort with an average diagnostic yield, some cases were excluded due to CT imaging conditions and inadequate follow-up. Therefore, the results of this study may not be generalizable and warrant external validation through prospective, multicenter cohort studies. Finally, the clinical significance of subdividing bronchus types belonging to negative CT-BS in rEBUS-TBB, which is highly dependent on the presence of CT-BS, was limited. This should be verified using newer guided bronchoscopic techniques, such as bronchoscopic transparenchymal nodule access and RAB, which is considered to be less dependent on the presence of CT-BS [35,40,41]. In addition, lesion visibility on fluoroscopy was also a key predictor of successful bronchoscopic diagnosis, regardless of CT-BS. Combinations of approaches such as cone-beam CT and augmented fluoroscopy as assistive guidance for lesions that are invisible on fluoroscopy may improve bronchoscopic diagnostic outcomes and further expand indications for bronchoscopy [42]. However, these newer high-accuracy guided bronchoscopic techniques usually require general anesthesia management, cost substantially more than rEBUS-TBB, which is performed under conscious sedation, and are not widely used in all countries. Not all patients require these newer techniques, as majority of PPLs in CT-BS group I can be detected by rEBUS and diagnosed, with a few exceptions such as type Ic. CT-BS subclassification will play an important role in maximizing the cost-effectiveness of these new and old modalities.

## 5. Conclusions

In conclusion, CT-BS subclassification may provide useful information to facilitate the selection of bronchoscopic techniques for accurate diagnosis of PPLs. rEBUS-TBB generally had an acceptable diagnostic performance in CT-BS group I. However, type IC is exceptional, and it may be useful to obtain larger samples with a 1.9 mm forceps (or consider cryoprobes). rEBUS-TBB is not expected to provide sufficient diagnostic performance in CT-BS group II; therefore, newer guided bronchoscopic techniques appropriate for each bronchus type should be considered.

## Figures and Tables

**Table 1:** Characteristics of patients and lesions in CT- group I.

	CT-BS Group I				
	All Types	Type Ia	Type Ib	Type Ic	*p*-Value
Characteristics	*N* = 792	*N* = 400	*N* = 287	*N* = 105	
Sex					<0.001
Male	462 (58.3)	262 (65.5)	152 (53.0)	48 (45.7)	
Female	330 (41.7)	138 (34.5)	135 (47.0)	57 (54.3)	
Age, median, years (range)	70 (17–90)	70 (17–90)	70 (35–89)	69 (38–85)	0.853
Lesion size, mm					<0.001
Median (range)	25.7 (7.0–100.2)	25.8 (7.0–90.3)	27.3 (9.5–100.2)	20.0 (8.9–81.1)	
≤20	244 (30.8)	115 (28.7)	73 (25.4)	56 (53.3)	
>20 to ≤30	273 (34.5)	143 (35.8)	93 (32.4)	37 (35.3)	
>30	275 (34.7)	142 (35.5)	121 (42.2)	12 (11.4)	
Lobar location					0.642
RUL/LUS	393 (49.6)	204 (51.0)	135 (47.0)	54 (51.4)	
RML/lingula	94 (11.9)	51 (12.8)	32 (11.1)	11 (10.5)	
RLL/LLL	305 (38.5)	145 (36.2)	120 (41.8)	40 (38.1)	
Lesion location from the hilum					0.662
Inner	202 (25.5)	107 (26.8)	68 (23.7)	27 (25.7)	
Outer	590 (74.5)	293 (73.2)	219 (76.3)	78 (74.3)	
Locational relationship with pleura					<0.001
Apart from the pleura	443 (55.9)	202 (50.5)	151 (52.6)	90 (85.7)	
Abutting on the pleura	349 (44.1)	198 (49.5)	136 (47.4)	15 (14.3)	
Lesion appearance on CT					<0.001
Solid	576 (72.7)	383 (95.8)	166 (57.8)	27 (25.7)	
Ground-glass	216 (27.3)	17 (4.2)	121 (42.2)	78 (74.3)	
Visibility on fluoroscopy					<0.001
Visible	609 (76.9)	335 (83.8)	212 (73.9)	62 (59.0)	
Invisible	183 (23.1)	65 (16.2)	75 (26.1)	43 (41.0)	
Final diagnosis					0.433
Malignant	660 (83.3)	332 (83.0)	236 (82.2)	92 (87.6)	
Benign	132 (16.7)	68 (17.0)	51 (17.8)	13 (12.4)	

Data are presented as *N* (%), unless otherwise stated. Abbreviations: CT-BS, computed tomography bronchus sign; RUL, right upper lobe; LUS, left upper segment; RML, right middle lobe; RLL, right lower lobe; LLL, left lower lobe; CT, computed tomography.

**Table 2:** Characteristics of patients and lesions in CT- group II.

	CT-BS Group II				
	All Types	Type IIa	Type IIb	Type IIc	*p*-Value
Characteristics	*N* = 229	*N* = 106	*N* = 95	*N* = 28	
Sex					0.340
Male	147 (64.2)	67 (63.2)	65 (68.4)	13 (46.4)	
Female	82 (35.8)	39 (36.8)	30 (31.6)	15 (53.6)	
Age, median, years (range)	70 (21–91)	70.5 (21–85)	69 (29–91)	72 (27–81)	0.930
Lesion size, mm					<0.001
Median (range)	15.6 (6.2–98.1)	18.8 (6.9–98.1)	13.9 (6.2–42.0)	13.0 (6.5–28.9)	
≤20	160 (69.9)	58 (54.7)	78 (82.1)	24 (85.7)	
>20 to ≤30	35 (15.3)	20 (18.9)	11 (11.6)	4 (14.3)	
>30	34 (14.8)	28 (26.4)	6 (6.3)	0 (0)	
Lobar location					0.149
RUL/LUS	110 (48.0)	52 (49.1)	48 (50.5)	10 (35.7)	
RML/lingula	33 (14.4)	18 (17.0)	8 (8.4)	7 (25.0)	
RLL/LLL	86 (37.6)	36 (34.0)	39 (41.1)	11 (39.3)	
Lesion location from the hilum					<0.001
Inner	88 (38.4)	63 (59.4)	14 (14.7)	11 (39.3)	
Outer	141 (61.6)	43 (40.6)	81 (85.3)	17 (60.7)	
Locational relationship with pleura					<0.001
Apart from the pleura	172 (70.2)	83 (78.3)	49 (51.6)	24 (85.7)	
Abutting on the pleura	73 (29.8)	23 (21.7)	46 (48.4)	4 (14.3)	
Lesion appearance on CT					0.007
Solid	186 (81.2)	95 (89.6)	72 (75.8)	19 (67.9)	
Ground-glass	43 (18.8)	11 (10.4)	23 (24.2)	9 (32.1)	
Visibility on fluoroscopy					0.021
Visible	133 (54.3)	68 (64.2)	51 (53.7)	10 (35.7)	
Invisible	112 (45.8)	38 (35.8)	44 (46.3)	18 (64.2)	
Final diagnosis					0.846
Malignant	180 (78.6)	85 (80.2)	73 (76.8)	22 (78.6)	
Benign	49 (21.4)	21 (19.8)	22 (23.2)	6 (21.4)	

Data are presented as No. (%) unless otherwise stated. Abbreviations: CT-BS, computed tomography bronchus sign; RUL, right upper lobe; LUS, left upper segment; RML, right middle lobe; RLL, right lower lobe; LLL, left lower lobe; CT, computed tomography.

**Table 3:** Procedural details for all bronchus types.

	CT-BS Group I				CT-BS Group II			
	Type Ia	Type Ib	Type Ic	*p*-Value	Type IIa	Type IIb	Type IIc	*p*-Value
Characteristics	*N* = 400	*N* = 287	*N* = 105		*N* = 106	*N* = 95	*N* = 28	
rEBUS finding				0.082				0.003
Within	241 (60.2)	177 (61.7)	49 (46.7)		25 (23.6)	34 (35.8)	7 (25.0)	
Adjacent to	135 (33.8)	91 (31.7)	43 (41.0)		61 (57.5)	32 (33.7)	9 (32.1)	
Invisible	24 (6.0)	19 (6.6)	13 (12.4)		20 (18.9)	29 (30.5)	12 (42.9)	
GS				<0.001				<0.001
Thin GS	179 (44.8)	71 (24.7)	14 (13.3)		25 (23.6)	44 (46.3)	5 (17.9)	
Thick GS	130 (32.4)	124 (43.2)	35 (33.4)		22 (20.7)	6 (6.3)	0 (0)	
Non-GS	91 (22.8)	92 (32.1)	56 (53.3)		59 (55.7)	45 (47.4)	23 (82.1)	
Forceps size	(*N* = 379)	(*N* = 285)	(*N* = 105)	<0.001	(*N* = 93)	(*N* = 90)	(*N* = 24)	0.004
1.9 mm	220 (55.0)	215 (74.9)	91 (86.7)		68 (64.2)	47 (49.5)	19 (67.9)	
1.5 mm	179 (44.8)	70 (24.4)	14 (13.3)		25 (23.6)	43 (45.3)	5 (17.9)	
TBNA	42 (10.5)	34 (11.8)	15 (14.3)	0.541	53 (50.0)	20 (21.1)	13 (46.4)	<0.001
Brushing	326 (81.5)	203 (70.7)	48 (45.7)	<0.001	47 (44.3)	49 (51.6)	8 (28.6)	<0.001
Bronchial lavage	7 (1.8)	8 (2.8)	0 (0)	0.192	2 (1.9)	1 (1.1)	0 (0)	0.707

Data are presented as *N* (%), unless otherwise stated. Abbreviations: CT-BS, computed tomography bronchus sign; rEBUS, radial endobronchial ultrasound; GS, guide sheath; TBNA, transbronchial needle aspiration.

**Table 4:** Logistic regression analysis of factors affecting successful diagnosis in CT-BS group I.

Variables	Total *N* = 792	Diagnostic Cases (%) *n* = 601 (75.9)	Univariable Analysis		Multivariable Analysis	
			OR (95% CI)	*p*-Value	Adjusted OR (95% CI)	*p*-Value
Sex						
Male	462	359 (77.7)	1.27 (0.91–1.76)	0.157		
Female	330	242 (73.3)	1.00			
Age, years						
>70	369	281 (76.2)	1.03 (0.74–1.42)	0.869		
≤70	423	320 (75.7)	1.00			
Lesion size, mm						
>20	548	453 (82.7)	3.09 (2.20–4.34)	<0.001	1.86 (1.27–2.72)	0.001
≤20	244	148 (60.7)	1.00		1.00	
Lobar location						
RUL/LUS	393	300 (76.3)	1.05 (0.76–1.45)	0.768		
Others	399	301 (75.4)	1.00			
Lesion location from the hilum						
Inner	202	164 (81.2)	1.51 (1.91–2.25)	0.042	1.44 (0.92–2.24)	0.108
Outer	590	437 (74.1)	1.00		1.00	
Locational relationship with pleura						
Apart from the pleura	443	338 (76.3)	1.05 (0.76–1.46)	0.759		
Abutting on the pleura	349	301 (86.2)	1.00			
Lesion appearance on CT						
Solid	576	449 (78.0)	1.49 (1.05–2.12)	0.027	0.79 (0.51–1.25)	0.317
Ground-glass	216	152 (70.4)	1.00		1.00	
Visibility on fluoroscopy						
Visible	609	507(83.3)	4.71 (3.28–6.74)	<0.001	2.96 (1.98–4.42)	<0.001
Invisible	183	94 (51.4)	1.00		1.00	
Bronchus type						
Type Ia	400	320 (80.0)	2.53 (1.64–3.88) ^1^	<0.001 ^1^	1.78 (1.04–3.05) ^1^	0.035 ^1^
Type Ib	287	219 (76.3)				
Type Ic	105	62 (59.0)	1.00		1.00	
rEBUS finding						
Within	467	405 (86.7)	8.04 (4.47–14.50) ^2^	<0.001 ^2^	4.86 (2.58–9.14) ^2^	<0.001 ^2^
Adjacent to	269	178 (66.2)				
Invisible	56	18 (32.1)	1.00		1.00	
Guide sheath						
With	553	440 (79.6)	1.89 (1.34–2.65)	<0.001	1.49 (1.01–2.21)	0.044
Without	239	161 (67.4)	1.00		1.00	
TBNA						
Performed	91	73 (80.2)	1.33 (0.77–2.29)	0.305		
Not performed	701	528 (75.3)	1.00			

^1^ types Ia and Ib vs. Ic. ^2^ “Within” and “Adjacent to” vs. “Invisible”. Abbreviations: CT-BS, computed tomography bronchus sign; OR, odds ratio; CI confidence interval; RUL, right upper lobe; LUS, left upper segment; CT, computed tomography; rEBUS, radial endobronchial ultrasound; TBNA, transbronchial needle aspiration.

**Table 5:** Logistic regression analysis of factors affecting successful diagnosis in CT-BS group II.

Variables	Total *N* = 229	Diagnostic Cases (%) *n* = 110 (48.0)	Univariable Analysis		Multivariable Analysis	
			OR (95% CI)	*p*-Value	Adjusted OR (95%CI)	*p*-Value
Sex						
Male	147	74 (50.3)	1.30 (0.75–2.23)	0.350		
Female	82	36 (43.9)	1.00			
Age, years						
>70	113	59 (52.2)	1.39 (0.83–2.34)	0.212		
≤70	116	51 (44.0)	1.00			
Lesion size, mm						
>20	69	39 (56.5)	1.63 (0.92–2.88)	0.093	0.81 (0.40–1.65)	0.562
≤20	160	71 (44.4)	1.00		1.00	
Lobar location						
RUL/LUS	110	57 (51.8)	1.34 (0.80–2.25)	0.271		
Others	119	53 (44.5)	1.00			
Lesion location from the hilum						
Inner	88	47 (53.4)	1.06 (0.62–1.80)	0.843		
Outer	141	67 (47.5)	1.00			
Locational relationship with pleura						
Apart from the pleura	156	77 (49.4)	1.18 (0.68–2.06)	0.558		
Abutting on the pleura	73	33 (45.2)	1.00			
Lesion appearance on CT						
Solid	186	100 (53.8)	3.84 (1.79–8.24)	<0.001	2.64 (1.10–6.29)	0.029
Ground-glass	43	10 (23.3)	1.00		1.00	
Visibility on fluoroscopy						
Visible	129	79 (61.2)	3.52 (2.02–6.11)	<0.001	2.27 (1.19–4.36)	0.014
Invisible	100	31 (31.0)	1.00		1.00	
Bronchus type						
Type IIa	106	56 (52.8)	1.85 (1.09–3.13) ^1^	0.022 ^1^	1.17 (0.62–2.23) ^1^	0.623 ^1^
Type IIb	95	44 (46.3)				
Type IIc	28	10 (35.7)	1.00		1.00	
rEBUS finding						
Within	66	50 (75.8)	10.20 (4.58–22.90) ^2^	<0.001 ^2^	8.43 (3.65–19.40) ^2^	<0.001 ^2^
Adjacent to	102	52 (51.0)				
Invisible	61	8 (13.1)	1.00		1.00	
Guide sheath						
With	102	55 (53.9)	1.53 (0.91–2.59)	0.111		
Without	127	55 (43.3)	1.00			
TBNA						
Performed	86	45 (52.3)	1.32 (0.77–2.25)	0.314		
Not performed	143	65 (45.5)	1.00			

^1^ type IIa vs. IIb and IIc. ^2^ “Within” and “Adjacent to” vs. “Invisible”. Abbreviations: CT-BS, computed tomography bronchus sign; OR, odds ratio; CI confidence interval; RUL, right upper lobe; LUS, left upper segment; CT, computed tomography; rEBUS, radial endobronchial ultrasound; TBNA, transbronchial needle aspiration.

## Data Availability

The dataset supporting the conclusions of this study is presented within the article. A detailed clinical dataset is not available to protect the privacy and confidentiality of the research subjects.

## Supplementary Materials

The following supporting information can be downloaded at: https://www.mdpi.com/article/10.3390/diagnostics13061064/s1, Table S1: Final diagnoses.
