# Supplementary material for: Computed Tomography Bronchus Sign Subclassification during Radial Endobronchial Ultrasound-Guided Transbronchial Biopsy: A Retrospective Analysis
Source: Diagnostics (Basel). 2023 Mar 10;13(6):1064. doi: 10.3390/diagnostics13061064 (PMC10047045; doi:10.3390/diagnostics13061064)
Supplement: Supplementary file 1 [file diagnostics-13-01064-s001.zip › diagnostics-2218247-supplementary.pdf]

Table S1. Final diagnoses.

Final Diagnoses	Total (Diagnostic Cases) N (n) = 1,021 (711)	CT-BS Group I (Diagnostic Cases) N (n) = 792 (601)	CT-BS Group II (Diagnostic Cases) N (n) = 229 (110)
Malignant	840 (608)	660 (516)	180 (92)
Primary lung cancer	741 (550)	615 (484)	126 (66)
Adenocarcinoma	537 (393)	461 (358)	76 (35)
Squamous cell carcinoma	128 (96)	101 (80)	27 (16)
Small cell carcinoma	29 (24)	21 (18)	8 (6)
Non-small cell carcinoma	22 (19)	15 (14)	7 (5)
Pleomorphic carcinoma	8 (5)	4 (3)	4 (2)
Adenosquamous carcinoma	7 (6)	6 (5)	1 (1)
Large cell neuroendocrine carcinoma	6 (5)	4 (4)	2 (1)
Carcinoid	2 (0)	1 (0)	1 (0)
NOS	2 (2)	2 (2)	0 (0)
Metastatic carcinoma	84 (56)	38 (31)	46 (25)
Malignant lymphoma	7 (2)	4 (1)	3 (1)
Mucoepidermoid carcinoma	2 (0)	1 (0)	1 (0)
Malignant solitary fibrous tumor	1 (0)	0 (0)	1 (0)
Suspected malignancy	5 (0)	2 (0)	3 (0)
Benign	181 (103)	132 (85)	49 (18)
Inflammation	69 (22)	46 (16)	23 (6)
Pneumonia	22 (21)	20 (19)	2 (2)
Granuloma	20 (14)	13 (8)	7 (6)
Mycobacterial infection	15 (10)	14 (10)	1 (0)
Organizing pneumonia	14 (13)	14 (13)	0 (0)
Fibrosis	10 (6)	7 (5)	3 (1)
Fungal infection	8 (6)	6 (5)	2 (1)
Lung abscess	5 (4)	5 (4)	0 (0)
Hamartoma	3 (2)	1 (1)	2 (1)
Allergic bronchopulmonary aspergillosis	2 (2)	2 (2)	0 (0)
IgG4-related disease	2 (1)	2 (1)	0 (0)
Bronchial atresia	2 (0)	0 (0)	2 (0)
Epithelial heamangioendothelioma	1 (1)	1 (1)	0 (0)
Round atelectasis	1 (0)	1 (0)	0 (0)
Amyloidosis	1 (1)	0 (0)	1 (1)
Mixed squamous cell and glandular papilloma	1 (0)	0 (0)	1 (0)
Sclerosing hemangioma	1 (0)	0 (0)	1 (0)
Teratoma	1 (0)	0 (0)	1 (0)
Other benignity	3 (0)	0 (0)	3 (0)
